# Influence of management regime and harvest date on the forage quality of rangelands plants: the importance of dry matter content

**DOI:** 10.1093/aobpla/plw045

**Published:** 2016-08-02

**Authors:** Iris Bumb, Eric Garnier, Denis Bastianelli, Jean Richarte, Laurent Bonnal, Elena Kazakou

**Affiliations:** 1 Montpellier Supagro, Centre d’Ecologie Fonctionnelle et Evolutive (UMR CEFE), CNRS - Université de Montpellier - Université Paul-Valéry Montpellier - EPHE, Campus CNRS, 1919 Route de Mende, 34293 Montpellier Cedex 5, France; 2 Centre d’Ecologie Fonctionnelle et Evolutive (UMR CEFE), CNRS - Université de Montpellier - Université Paul-Valéry Montpellier - EPHE, Campus CNRS, 1919 Route De Mende, 34293 Montpellier Cedex 5, France; 3 CIRAD, UMR SELMET, Baillarguet, 34398 Montpellier Cedex 5, France

**Keywords:** Dry matter content, dry matter digestibility, fertilization, fibre content, grazing, growth form, nitrogen concentration, nutritional value of forage

## Abstract

In spite of their recognized ecological value, relatively little is known about the nutritional value of species-rich rangelands for herbivores. We investigated the sources of variation in dry matter digestibility (DMD), neutral detergent fibre content (NDF) and nitrogen concentration (NC) in plants from species-rich Mediterranean rangelands in southern France, and tested whether the dry matter content (DMC) was a good predictor of the forage quality of different plant parts. Sixteen plant species with contrasting growth forms (rosette, tussock, extensive and stemmed-herb) were studied, representative of two management regimes imposed in these rangelands: (i) fertilization and intensive grazing and (ii) non-fertilization and moderate grazing. Among the 16 plant species, four species were found in both treatments, allowing us to assess the intraspecific variability in forage quality and DMC across the treatments. The components of nutritional value (DMD, NDF and NC) as well as the DMC of leaves, stems and reproductive plant parts, were assessed at the beginning of the growing season and at peak standing biomass. All components of nutritional value and DMC were affected by species growth form: rosettes had higher DMD and NC than tussocks; the reverse being found for NDF and DMC. As the season progressed, DMD and NC of the different plant parts decreased while NDF and DMC increased for all species. DMC was negatively related to DMD and NC and positively to NDF, regardless of the source of variation (species, harvest date, management regime or plant part). Path analysis indicated that NDF was the main determinant of DMD. Better assessment of forage quality in species-rich systems requires consideration of their growth form composition. DMC of all plant parts, which is closely related to NDF, emerged as a good predictor and easily measured trait to estimate DMD in these species-rich systems.

## Abstract (precis)

We investigated the sources of variation in forage quality in plants from species-rich Mediterranean rangelands in southern France. Digestibility was affected by species growth form, harvest date, developmental stage and management regime, and differed between leaves, stems and reproductive parts. The dry matter content of the different plant parts, an estimate of the density of their tissues closely related to fibre content, emerged as a good predictor and an easily measured trait to estimate digestibility in the wide range of species spanned in our study.

## Introduction

Rangelands provide a range of ecosystem goods and services including fodder provision, soil stability, carbon sequestration and maintenance of species diversity, as well as water and climate regulation (De Bello *et al.* 2010; Yahdjian *et al.* 2015). The provision of fodder which is key to herbivore diet (cf. Lemaire *et al.* 2011), depends on such factors as the amount and seasonality of biomass production, forage quality and management flexibility (Duru *et al.* 2010; Duru *et al.* 2014*a*). Differences in management regimes pertaining to defoliation or fertilizer supply affect vegetation structure and function, leading to differences in these various components of the fodder provision (Garnier and Navas 2012).

In spite of their widely recognized ecological value, little is known about the nutritional value of species-rich rangelands for herbivores (Duru *et al.* 2008*a*). A better understanding requires improved knowledge of the nutritional value of the large number of plant species in these systems. Dry matter digestibility (DMD), which provides a synthetic measure of the amount of energy in plant constituents available for herbivores, especially ruminants, is a key component of this nutritional value (Bruinenberg *et al.* 2002).

Whole plant digestibility depends on several factors: (i) species, in particular its taxonomic affiliation (Pontes *et al.* 2007; Carrère *et al.* 2010) and growth form; (ii) plant developmental stage (Buxton 1996; Bruinenberg *et al.* 2002); (iii) management regimes, in particular fertilization (Duru *et al.* 2000; Pontes *et al.* 2007), and grazing intensity (Bardgett and Wardle 2003; Garcia *et al.* 2003). Fibre content (hemicellulose, cellulose and lignin) and nitrogen concentration (NC) have been shown to have significant effects on digestibility (Jung and Allen 1995; Karn *et al.* 2006). To date, only a few studies have assessed the relative importance of management regime and/or developmental stage on the digestibility of whole-plant or different plant parts across a wide range of species beyond grasses (Duru 1997; Calvière and Duru 1999; Pontes *et al.* 2007; Carrère *et al.* 2010).

The aim of the present study is to analyze the relative importance of the factors, species growth forms, plant development stages and management regimes, on digestibility, fibre content and nitrogen concentration through a trait-based approach to plant functioning. The use of plant traits which enables us to assess the interactions between organisms and their environment simultaneously on a large number of species, has been advocated as a relevant means to address pending questions in species-rich eco- and agro-systems (reviewed in Garnier and Navas 2012; Duru *et al.* 2014*a*). Differences in digestibility induced by one or several of the above-mentioned factors have been associated with a number of functional traits (Violle *et al.* 2007), either at the species (Al Haj Khaled *et al.* 2006; Pontes *et al.* 2007) or community level (Andueza *et al.* 2010; Gardarin *et al.* 2014). Among the traits tested in these previous studies, Leaf Dry Matter Content (LDMC: the ratio of leaf dry mass to water saturated fresh mass) was the most consistent and best predictor of digestibility and was positively related to fibre content (Al Haj Khaled *et al.* 2006) and negatively correlated with digestibility at both species (Louault *et al.* 2005; Al Haj Khaled *et al.* 2006; Pontes *et al.* 2007) and community levels (Duru *et al.* 2008*a*; Andueza *et al.* 2010; Gardarin *et al.* 2014). Further, previous studies have suggested that the LDMC of dominant species was a pivotal trait for grouping species into functional types to improve the assessment of digestibility in species-rich rangelands (Ansquer *et al.* 2004; Al Haj Khaled *et al.* 2006; Duru *et al.* 2008*a*).

So far, trait-digestibility relationships have mostly been established for grass species which represent only a fraction of the species present in species-rich rangelands. Only one study has considered more than one forb species (Louault *et al.* 2005). Further, since digestibility (DMD) and dry matter content (DMC) differ among plant parts, relationships between these two variables should be considered separately for each plant part. We therefore addressed the following question: Does DMC adequately capture differences in digestibility and components of forage quality among plant species from species-rich rangelands across different management regimes and developmental stages? If yes, is this pattern validated between different plant parts?

Our first objective was to test the influence of different factors on dry matter digestibility (DMD) and components of forage quality in different plant parts. We hypothesized that fertilization and intense grazing would favour species with high NC and/or low DMC (reviewed in Garnier *et al.* 2016) and low fibre content and thus high DMD. We predicted that these effects would be similar within species but would vary according to growth form, species and developmental stage. Our second objective was to determine the influence of DMC and other components of forage quality on DMD. We hypothesized that DMC would co-vary with DMD and that this relationship would be maintained for each plant part. We also assessed the relative influence of the above-mentioned factors on DMD. Our study included 16 plant species chosen as representative of those found in a Mediterranean rangeland in southern France, and spanning the range of growth forms in these systems.

## Methods

### Study site and experimental design

The study was conducted using abundant plant species found in dry calcareous rangelands in southern France located on a limestone plateau (Larzac Causse) at the La Fage research station of INRA (French National Institute for Agricultural Research) (43°55′N, 3°05′E, 790 m above sea level). The climate of the plateau is sub-humid with a Mediterranean influence, with cool, wet winters and warm dry summers. Annual mean precipitation of 1070 mm occurs mainly during spring and autumn, and mean monthly temperatures vary from 1 °C in January to 19 °C in August (data 1973–2013: from the on-site meteorological station; see Chollet *et al.* (2014)). The vegetation is dominated by perennial herbaceous species, the most abundant being *Bromopsis erecta*, along with loosely scattered shrubs and trees of which *Buxus sempervirens* and *Juniperus oxycedrus* are two of the most abundant shrub and tree species (Bernard 1996; Bernard-Verdier *et al.* 2012; Barkaoui *et al.* 2013; Chollet *et al.* 2014). Since 1972, the station’s 280 ha of rangelands have been grazed by a sheep flock (Romane breed) raised outdoors year-round for meat production.

The INRA experimental station is divided into paddocks that vary from 4.7 to 24.5 ha which are subjected to two different management regimes. We selected six of these paddocks (three per management regime) which were close to one another and comparable in terms of soil composition and topography. The first management regime consisted of fertilization and grazing (“G + F+” treatment hereafter) of 0.61 kg kg ^−^ ^1^ (proportion of total annual biomass produced that is removed by grazing; with intense grazing in spring). In this first treatment, mineral nitrogen (65 kg ha ^−^ ^1^ year) and phosphorus (40 kg ha ^−^ ^1^ every three years) had been added since 1978. The second management regime applied since 1972 consisted of moderate grazing starting in early May of 0.20 kg kg ^−^ ^1^, without fertilization (“G + F−” treatment hereafter). Within each of the six selected paddocks, three plots (plot area comprised between 200 and 500 m^2^ to account for differences in species density), were set for the present study making a total of 9 plots for each treatment.

### Species studied and sampling

Based on botanical surveys of the paddocks (Bernard-Verdier *et al.* 2012; Barkaoui *et al.* 2013; Chollet *et al.* 2014), we selected 16 plant species representing three growth forms (Table 1), among those most abundant in the two treatments: six species were present only in G + F−, six species were present only in G + F+ and four were present in both treatments (*Bromopsis erecta, Pilosella officinarum*, *Potentilla tabernaemontani* and *Poterium sanguisorba*) (Table 1).

**Table 1.** List of species studied, with abbreviation, location in each treatment, taxonomic group, life cycle and growth form (Pérez-Harguindeguy *et al*. 2013). G + F−: non-fertilized and moderately grazed treatment; G + F+: fertilized and intensely grazed treatment.

Species	Abbrev.	Treatment		Taxonomic group	Life cycle	Growth form
*Anthyllis vulneraria*	Av	G+F−		Fabaceae	Perennial/Annual	Rosette
*Brachypodium pinnatum*	Bp	G+F−		Poaceae	Perennial	Tussock
*Carex humilis*	Ch	G+F−		Cyperaceae	Perennial	Tussock
*Helianthemum apenninum*	Ha	G+F−		Cistaceae	Perennial	Extensive and stemmed-herb
*Helianthemum canum*	Hc	G+F−		Cistaceae	Perennial	Extensive and stemmed-herb
*Stipa pennata*	Sp	G+F−		Poaceae	Perennial	Tussock
*Capsella bursa-pastoris*	Cb		G+F+	Brassicaceae	Annual	Rosette
*Erodium cicutarium*	Ec		G+F+	Geraniaceae	Annual	Rosette
*Geranium molle*	Gm		G+F+	Geraniaceae	Annual	Extensive and stemmed-herb
*Plantago lanceolata*	Pl		G+F+	Plantaginaceae	Perennial	Rosette
*Poa bulbosa*	Pb		G+F+	Poaceae	Perennial	Tussock
*Veronica arvensis*	Va		G+F+	Plantaginaceae	Annual	Extensive and stemmed-herb
*Bromopsis erecta*	Be	G+F−	G+F+	Poaceae	Perennial	Tussock
*Pilosella officinarum*	Po	G+F−	G+F+	Asteraceae	Perennial	Rosette
*Potentilla tabernaemontani*	Pt	G+F−	G+F+	Rosaceae	Perennial	Extensive and stemmed-herb
*Poterium sanguisorba*	Ps	G+F−	G+F+	Rosaceae	Perennial	Rosette

In spring 2013, biomass was harvested at two times chosen on the basis of the grazing calendar and plant phenology, which was delayed in the G + F− treatment due to the absence of fertilization in this treatment (Chollet *et al.* 2014): the first harvest occurred at the beginning of vegetative growth (mid-April for G + F+ and end-of-April to early-May for G + F−) when sheep grazed for the first time, and the second occurred at the peak biomass (end-of-May for G + F+ and mid-June for G + F−).

Two batches of plant samples were collected. The first batch was used to assess forage quality measurements. Thirty to 150 individuals without apparent grazing damage were collected for each plant species in each plot, so that enough biomass per species was available to conduct subsequent analyses. The samples were placed into plastic bags with tissues moistened with deionized water. Individuals of each plant species per plot were combined together and kept in a cooling-box until return to the laboratory. Each combined sample was then sorted into leaves (lamina and sheath), stems and reproductive parts (flowers and fruits), and dried at 60 °C for 72 h. Samples were ground using a ZM100 centrifugal mill through a 1 mm screen. The second batch of samples was used for DMC measurements for all the 16 species. DMC was measured on the above-ground parts of at least 12 individuals per species in each plot. After harvesting these individuals were immediately placed in a test tube with water and placed in a cool box. The tubes were then stored at 4 °C for at least 6 h to ensure full rehydration of the samples (cf. Garnier *et al.* 2001).

### Digestibility and components of forage quality measurements

DMD and components of forage quality (NC and NDF) were assessed from the first batch, which included 469 plant part samples of the 16 species collected from the 18 plots from the two management regimes and the two harvest dates.

Near infrared reflectance spectroscopy (NIRS) is a non-destructive and highly precise physical method based on selective absorption of near infrared electromagnetic waves by organic molecules (Birth and Hecht 1987). NIRS has proved useful to relate the spectra of samples to their laboratory biochemical values in a number of digestibility studies (Aufrère *et al.* 1996; Stuth *et al.* 2003; Andrés *et al.* 2005). In the present study, dried and ground samples of different plant parts of each species from each plot were analyzed using NIRS. Reflectance spectra were collected from duplicate samples presented in ring cells equipped with quartz glass using a FOSS Nirsystem 6500 spectrometer (FOSS Nirsystems, Silver Spring, MD, USA) operating at 400–2500 nm so as to produce an average spectrum with 870–1013 data points. Existing calibrations at CIRAD (French International Centre of Agricultural Research for Development) between the spectral properties and the measured digestibility were updated and adapted to our samples with reference to spectral analyses from 24 samples representative of our dataset. Calibration was performed using modified partial least square regression with WINISI software (version 4, Infrasoft International, Port Matilda, PA, USA). Statistical parameters of the calibration models for forage quality measurements are presented in File 1 [**See Supporting Information**]. As measured and predicted digestibility were strongly correlated [**See Supporting Information**], the NIRS method was used to predict digestibility from spectral data.

Chemical parameters known to be related to the nutritional value of the samples were also measured in the 24 samples used for calibrations: *in vitro* DMD (g kg ^−^ ^1^) was measured by the pepsin-cellulase method of Aufrère *et al.* (2007); total NC (g kg ^−^ ^1^) was measured by the Kjeldahl method and fibre content (Neutral Detergent Fibre, NDF %) was measured by the Van Soest sequential detergent method with an amylase and protease pre-treatment (Van Soest *et al.* 1991).

### Dry matter content measurements

DMC was measured from the second batch of samples. After rehydration, plant parts were sorted into three components. First, the youngest mature leaf was cut at the petiole insertion for dicotyledons and at the lamina for monocotyledons (including the sheath for Poaceae); second, the entire stem of one individual was included (or sometimes a portion); and third, the reproductive parts which were a mix of flowers, fruits and peduncles. DMC was assessed for each of these component plant parts for the 12 individuals per species collected in each plot, according to the protocol described in Garnier *et al.* (2001). Each plant part was gently blotted dry before weighing to obtain its water-saturated fresh mass, dried for 72 h at 60 °C and weighed to measure dry mass. DMC was calculated as the ratio between the dry mass of the plant part and its water-saturated fresh mass.

### Data analysis

Statistical analyses were performed using R software (R Development Core Team 2010). Linear models were used to test the influence of management regime, harvest date, growth form, and species (nested within a growth form) and their interactions with DMD, components of forage quality and DMC. These analyses were performed for each plant part (leaf, stem and reproductive part) of the studied species. The model selection, based on ANOVA, used manual backward elimination and stepwise regression with the ‘lm’ function. The normality of the distribution of residuals was verified for each model. The same procedure was applied to the four plant species in both treatments to test the influence of management regime, harvest date, and species and their interactions for each plant part.

Principal Component Analysis (PCA) was performed for each plant part at the time of peak biomass (harvest date 2) using the ‘lme4’ package to evaluate differences in DMD, the other components of forage quality and the DMC between species growth forms. The projection of the points on axis 1 was recovered to test differences in DMD, other forage quality components and DMC between growth forms using one-way ANOVA, followed by a Tukey test to determine differences between plant parts at the time of peak biomass.

A variance decomposition analysis was performed for the four plant species present in both treatments in order to assess intraspecific variability of leaf DMD, components of forage quality and DMC (see Table 1) (Xu 2003).

Pearson and Spearman correlations were calculated for each plant part of the 16 species, between forage quality and DMC. Intercepts and slopes were tested using ANCOVA to compare the influences of developmental stage and plant parts.

Path analysis was used to understand direct and indirect causal relationships among variables (management regime, harvest date, DMD, components of forage quality and DMC) *via* a combination of linear models forming a correlation network (Shipley 2000). This method, based on Structural Equation Modelling, could be used to quantify the relative importance of different factors (management regime and harvest date) on DMD, components of forage quality and DMC for each plant part. It also allows to identify the best combination of traits or components of forage quality predicting variation in DMD. DMD, NC, NDF and DMC were scaled with the ‘scale’ function from the ‘base’ package (Becker *et al.* 1988), and the ‘species’ effect was added as a random factor in the models tested. Several initial global conceptual models were developed following our hypothesis of causal relationships between the different variables (fertilization, grazing, DMD, NC, NDF and DMC), involving variations in direct and indirect effects between those variables. The overall fits of the path models were tested with a generalized d-sep test (Shipley 2009) using the ‘ggm’ package in R. A Fisher’s C test of the overall models was performed between each initial overall model proposed (expected covariance structure) and the data measured (observed covariance structure) in order to select the best model. A chi-squared distribution and a non-significant *P* value of fit indicated that the initial model structure and the data did not differ significantly and that the model suitably represented the data. The significance of regression path coefficients associated with each single headed arrow, representing causal relationships between variables, was also tested. The estimates of each linear model were used to indicate the relative strength and influence of each of the relationships in the model. By convention, standardized regression path coefficients (i.e. estimate) > 0.8 are considered to have a large influence, those equal to 0.5 moderate influence, and those < 0.2 little influence (see Shipley (2009) for more details on the method). Indirect effects of factors on DMD were calculated by multiplying all coefficients encountered on the paths linking variables and by summing them if there were multiple paths (Shipley 2000).

## Results

### Interspecific variation in forage quality and dry matter content

Our data showed large variation in DMD of leaves between harvest dates 1 and 2 (means per species ranged from 408 to 892 g kg ^−^ ^1^ for the 16 studied species).

**Figure 1.**
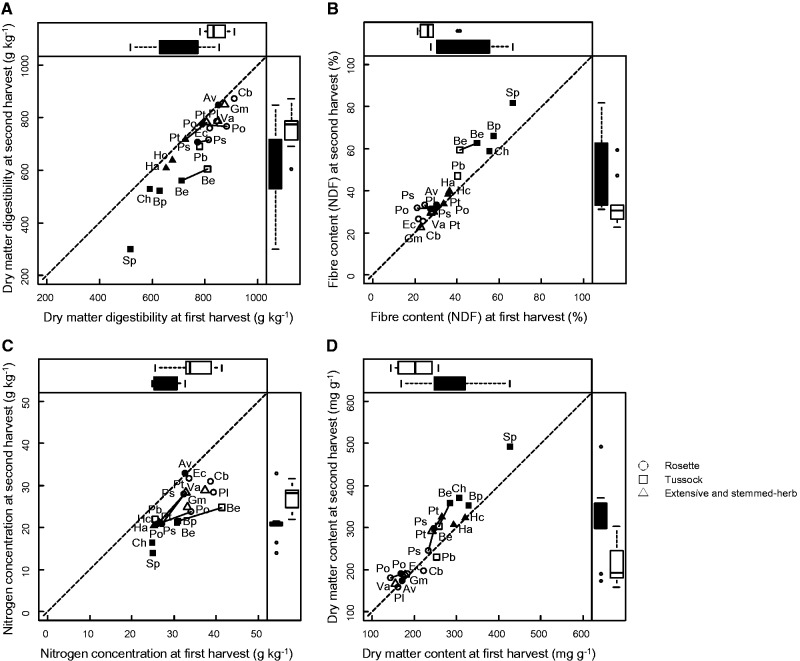
Influence of management regimes (grazed and fertilized (G + F+) and grazed and non-fertilized (G + F−) treatments) and harvest dates on leaves: (A) dry matter digestibility (DMD), (B) fibre content (NDF), (C) nitrogen concentration (NC) and (D) dry matter content (DMC). Boxes and whisker plots at the top of the plots show the distribution of values at date 1 (the open circles represent outliers); Boxes and whisker plots at the right of the plots show the distribution of values at date 2 (the open circles represent outliers). Solid symbols and whisker plots represent the G + F− treatment, while open symbols and whisker plots represent the G + F+ treatment. Solid lines connect values for the four species found in both treatments. Species abbreviations are provided in Table 1.

At the beginning of growth (harvest date 1), the leaves were on average 10.4% more digestible and 25.6% richer in nitrogen than at the peak biomass (harvest date 2) (Fig. 1A, C and Table 2). *Stipa pennata* had the largest decrease in leaf DMD between the two dates (−53.2%), while *Anthyllis vulneraria* had the smallest decrease (−0.77%) (Fig. 1A). Leaf NDF and DMC were higher at the peak biomass (harvest date 2) (+14.7% and +9.7%, respectively) (Fig. 1B, D and Table 2). Stems and reproductive plant parts followed the same pattern as leaves between harvest dates (Table 2). In general, the decrease in DMD between harvests was more pronounced for species with the lowest DMD values (mostly true for leaves for which all data were available at both harvest dates) like *Stipa pennata*.

**Table 2.** Influence of harvest date, growth form, species, and management regime and their interactions on digestibility, other components of herbage quality (nitrogen concentration and fibre content) and dry matter content. Plant parts (leaf, stem and reproductive parts) were analyzed at the interspecific level. Only factors or interactions with indicators of significance (including “ns”) were selected in the model. Numbers represent the F value from ANOVA. Degrees of freedom are in brackets (df_factor_/df_residual_).

	Dry Matter Digestibility (DMD)			Nitrogen Concentration (NC)			Fibre content (NDF)			Dry Matter Content (DMC)		
	Leaf (*n = *229)	Stem (*n = *131)	Repro. parts (*n = *137)	Leaf (*n = *229)	Stem (*n = *131)	Repro. parts (*n = *137)	Leaf (*n = *229)	Stem (*n = *131)	Repro. parts (*n = *137)	Leaf (n = 229)	Stem (*n = *144)	Repro. parts (*n = *137)
Harvest date	489***_(1/169)_	395***_(1/84)_	299***_(1/80)_	620***_(1/137)_	311***_(1/76)_	619***_(1/50)_	443***_(1/172)_	816***_(1/84)_	530***_(1/71)_	190***_(1/175)_	23.0***_(1/111)_	19.0***_(1/104)_
Growth form	780***_(2/169)_	496***_(2/84)_	270***_(2/80)_	52.9***_(2/137)_	39.0***_(2/76)_	110***_(2/50)_	3543***_(2/172)_	1558***_(2/84)_	1097***_(2/71)_	904***_(2/175)_	90.3***_(2/111)_	125***_(2/104)_
Species	71.6***_(2/104)_	61.2***_(11/84)_	66.2***_(11/80)_	29.5***_(13/137)_	14.1***_(11/76)_	68.6***_(11/50)_	71.3***_(13/172)_	62.8***_(11/84)_	81.0***_(11/71)_	110***_(13/175)_	34.5***_(13/111)_	11.9***_(13/104)_
Management regime	1386***_(1/169)_	981***_(1/84)_	269***_(1/80)_	525***_(1/137)_	186***_(1/76)_	570***_(1/50)_	1644***_(1/172)_	572***_(1/84)_	49.4***_(1/71)_	1831***_(1/175)_	224***_(1/111)_	80.9***_(1/104)_
Harvest date × Management regime	*3.4*_(1/169)_	16.5***_(1/84)_		7.50**_(1/137)_	26.8***_(1/76)_	ns _(1/50)_	ns _(1/172)_	ns _(1/84)_	ns _(1/71)_	59.4***_(1/175)_		6.16*_(1/104)_
Harvest date × Growth form	83.3***_(2/169)_	18.4***_(1/84)_	8.7***_(2/80)_	23.3***_(2/137)_	10.2**_(2/76)_	4.31*_(2/50)_	110***_(2/172)_	18.2***_(1/84)_	23.0***_(2/71)_	11.7***_(2/175)_	ns _(1/111)_	7.97***_(2/104)_
Harvest date × Species	8.3***_(13/169)_	10.2***_(3/84)_		7.67***_(13/137)_	*2.33*_(3/76)_	3.21*_(4/50)_	11.8***_(13/172)_	10.7***_(3/84)_	8.49***_(4/71)_	8.70***_(13/175)_	6.67***_(4/111)_	5.23**_(3/104)_
Management regime × Growth form	*2.8*_(2/169)_	23.5***_(2/84)_	ns _(2/80)_	ns _(2/137)_		*3.01*_(2/50)_	*3.72*_(2/172)_		ns _(2/71)_			

Notes: ns, not significant;

* *P <* 0.05;

** *P <* 0.01;

*** *P <* 0.001;

marginally significant results (0.05 < *P <* 0.1) are shown in italics.

Across all the species, leaves, in general, had the highest DMD and NC and stems the lowest (3.17 < F_2,52 _< 22.4, *P *≤* *0.05). Reproductive plant parts had DMD values between those of leaves and stems (mean per plant part at harvest date 2: leaves = 69.0 g  kg ^−^ ^1^, reproductive parts = 65.2 g  kg ^−^ ^1^, stems = 58.0 g  kg ^−^ ^1^) and an NC equivalent to that of leaves (mean per plant part at harvest date 2: leaves = 24.1 g kg ^−^ ^1^, reproductive parts = 22.5 g kg ^−^ ^1^, stems = 14.0 g kg ^−^ ^1^). No significant difference was detected between plant parts in NDF (F_2,52 _= 1.61^ns^, *P *>* *0.05) or DMC (F_2,55 _= 1.31^ns^, *P *>* *0.05) (Fig. 1).

Fertilization increased leaf DMD and NC in all species (+19.8% and +24.1% increase, respectively) (Fig. 1A, C and Table 2). Conversely, NDF and DMC of leaves in the G + F+ treatment were lower than those in the G + F− treatment (−37.1% and −35.8%, respectively) (Fig. 1B, D and Table 2).

**Figure 2.**
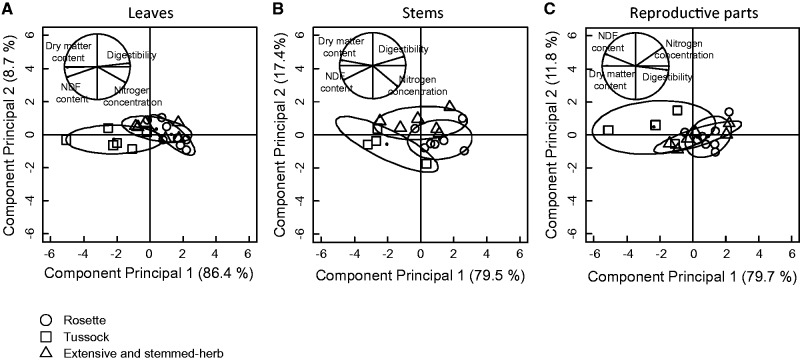
Principal component analysis between forage quality (dry matter digestibility (DMD), fibre content (NDF), and nitrogen concentration (NC)) and dry matter content (DMC) measured at the biomass peak (harvest date 2) for (A) leaves, (B) stems and (C) reproductive parts. Ellipses were drawn at a 0.70 confidence level around the points for each growth form.

Forage quality and DMC varied according to species growth form (Table 2; *P *≤* *0.05). These variations were identified in the PCA where the first axis accounted for 86.4% for leaves, 79.5% for stems, and 79.7% for reproductive parts (Fig. 2). Axis 1 was determined by DMD and NC opposed to NDF and DMC. Loading of species on axis 1 varied significantly among growth forms at the biomass peak. All the plant parts of species with the tussock growth form had lower DMD and higher NDF than the rosette and extensive and stemmed-herbs species at both harvest dates (Fig. 2; Tukey test). Plant species with differing growth forms responded differently to harvest date (Table 2). Tussocks had a higher decrease in DMD during plant growth (−22.9%) than rosettes (−7.34%) and extensive and stemmed-herbs (−4.60%). Tussock species with high NDF and DMC, such as *Stipa pennata*, had the highest decrease in DMD between harvest dates while DMD of species with low NDF and high NC, such as *Anthyllis vulneraria*, did not vary between harvest dates.

### Intraspecific variations in forage quality and dry matter content

**Figure 3.**
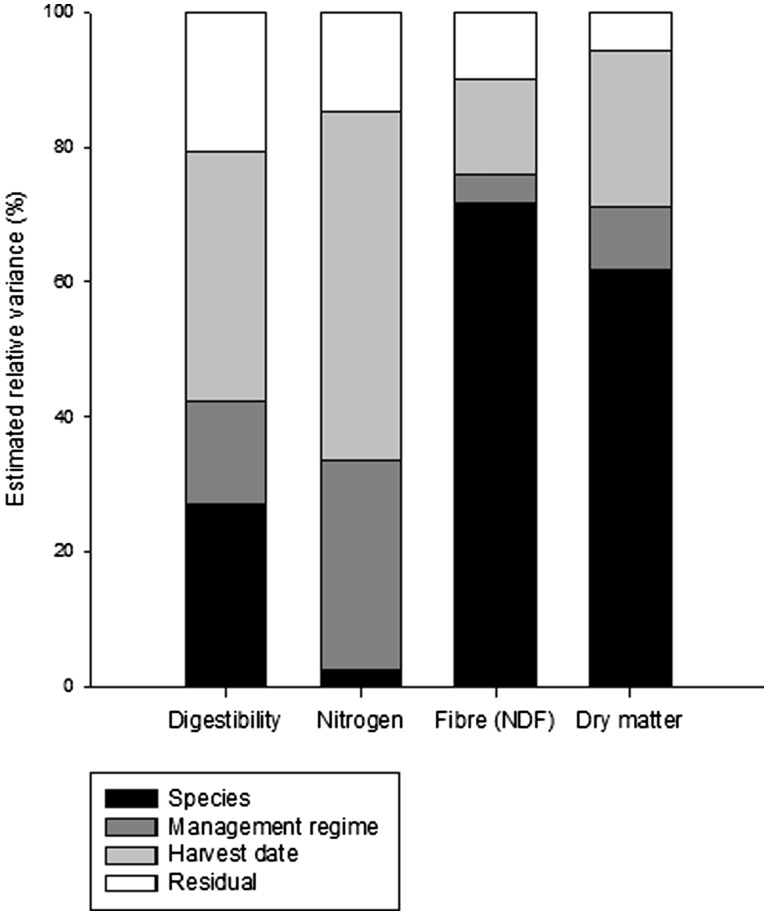
Estimated variance decomposition among species, management regime and developmental stage for forage quality (dry matter digestibility (DMD), fibre content (NDF), and nitrogen concentration (NC)) and dry matter content (DMC) of leaves.

For the four species grown in both treatments (fertilized and non-fertilized), we observed a decrease in DMD and NC (Fig. 1A, C) and an increase in NDF and DMC between harvest dates (Fig. 1B, D). Fertilization consistently induced an increase in DMD and NC (Fig. 1A, C) and a decrease in NDF and DMC (Fig. 1B, D). The relative influence of species, harvest date and fertilization on forage quality and DMC varied (Fig. 3). Considering leaf DMD, 27.1% of the variance was attributed to plant species, 37.0% to species developmental stage and 15.3% to the management regime (Fig. 3). For leaf NDF and DMC, more than 60% of the variance was attributed to species (71.8% and 61.7%, respectively). For leaf NC, little variance was attributed to species (2.40%), while most of the variance was attributed to developmental stage (51.5%) (Fig. 3).

### Relationships between dry matter content and forage quality

**Figure 4.**
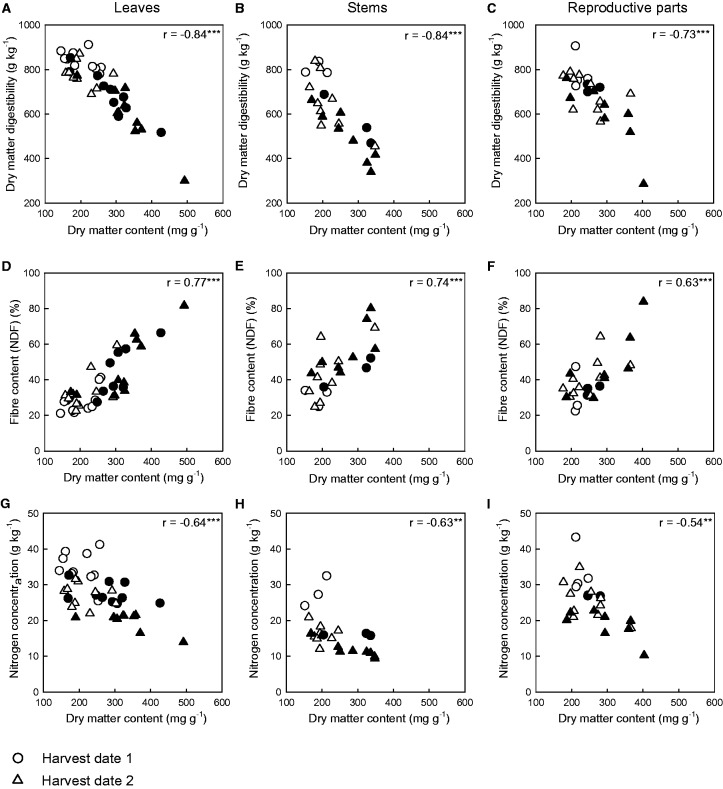
Relationships between forage quality and dry matter content of species plant parts: dry matter digestibility (DMD) and dry matter content (DMC) for (A) leaves, (B) stems and (C) reproductive plant parts; fibre content (NDF) and dry matter content (DMC) for (D) leaves, (E) stems and (F) reproductive plant parts; and nitrogen concentration (NC) and dry matter content (DMC) for (G) leaves, (H) stems and (I) reproductive plant parts. The r values are Pearson or Spearman correlation coefficients for all data. Solid symbols represent the G + F− treatment and open symbols represent the G + F+ treatment. See File 3 for correlation results at each harvest date **[see Supporting Information]**. Notes: ns, not significant; *P < 0.05; **P < 0.01; ***P < 0.001.

Results showed clear links between forage quality and DMC (Fig. 2 and File 2 [**see Supporting Information**]). Leaf DMC was negatively related to DMD (Fig. 4A) and NC (Fig. 4G) and positively related to leaf NDF (Fig. 4D). The same patterns were found for stems (Fig. 4B, E, H) and reproductive plant parts (Fig. 4C, F, I). Slopes of relationships between DMC and DMD or components of forage quality considering each plant part separately were similar for both harvest dates (Table 3). Intercepts of species’ developmental stages differed for DMD and NC for each plant part (Table 3), for which the intercept at harvest date 1 was higher than that at harvest date 2 (Table 3 and Fig. 4). The intercepts of the relationships between dry matter content and NDF were similar for leaves and reproductive plant parts (Table 3). The slopes of the relationships between DMD and DMC on the one hand, and DMD and components of forage quality on the other, were similar for the different plant parts (Table 3). Intercepts for the leaves were higher than those for the other plant parts (Table 3 and Fig. 4).

**Table 3.** Harvest date and plant parts influence on the slopes and intercepts of the relationships between dry matter content (DMC) and components of forage quality (dry matter digestibility (DMD), nitrogen concentration (NC) and fibre content (NDF)) presented in fig. 4. Numbers represent the F value from ANCOVA. Degrees of freedom are in brackets (df_factor_/df_residual_).

Relationship with the Dry Matter Content (DMC)		Harvest date		Plant parts	
		Slope	Intercept	Slope	Intercept
Dry Matter Digestibility (DMD)	Leaf	$0.17_{\text{(1/36)}}^{\text{ns}}$	5.62*_(1/36)_	$1.55_{\text{(2/82)}}^{\text{ns}}$	29.7***_(2/82)_
	Stem	$0.00_{\text{(1/19)}}^{\text{ns}}$	7.89*_(1/19)_		
	Reproductive parts	$0.00_{\text{(1/21)}}^{\text{ns}}$	*3.10*_(1/21)_		
Nitrogen Concentration (NC)	Leaf	$0.04_{\text{(1/36)}}^{\text{ns}}$	22.8***_(1/36)_	$0.58_{\text{(2/82)}}^{\text{ns}}$	44.1***_(2/82)_
	Stem	$0.40_{\text{(1/19)}}^{\text{ns}}$	23.1***_(1/19)_		
	Reproductive parts	$1.09_{\text{(1/21)}}^{\text{ns}}$	9.67**_(1/21)_		
Fibre Content (NDF)	Leaf	$0.00_{\text{(1/36)}}^{\text{ns}}$	$0.47_{\text{(1/36)}}^{\text{ns}}$	$0.11_{\text{(2/82)}}^{\text{ns}}$	10.1***_(2/82)_
	Stem	$0.91_{\text{(1/19)}}^{\text{ns}}$	6.10*_(1/19)_		
	Reproductive parts	$0.31_{\text{(1/21)}}^{\text{ns}}$	$1.75_{\text{(1/21)}}^{\text{ns}}$		

Notes: ns, not significant;

*P < 0.05;

**P < 0.01;

***P < 0.001;

marginally significant results (0.05 < *P <* 0.1) are shown in italics.

### Relative influence of factors affecting dry matter digestibility

**Figure 5.**
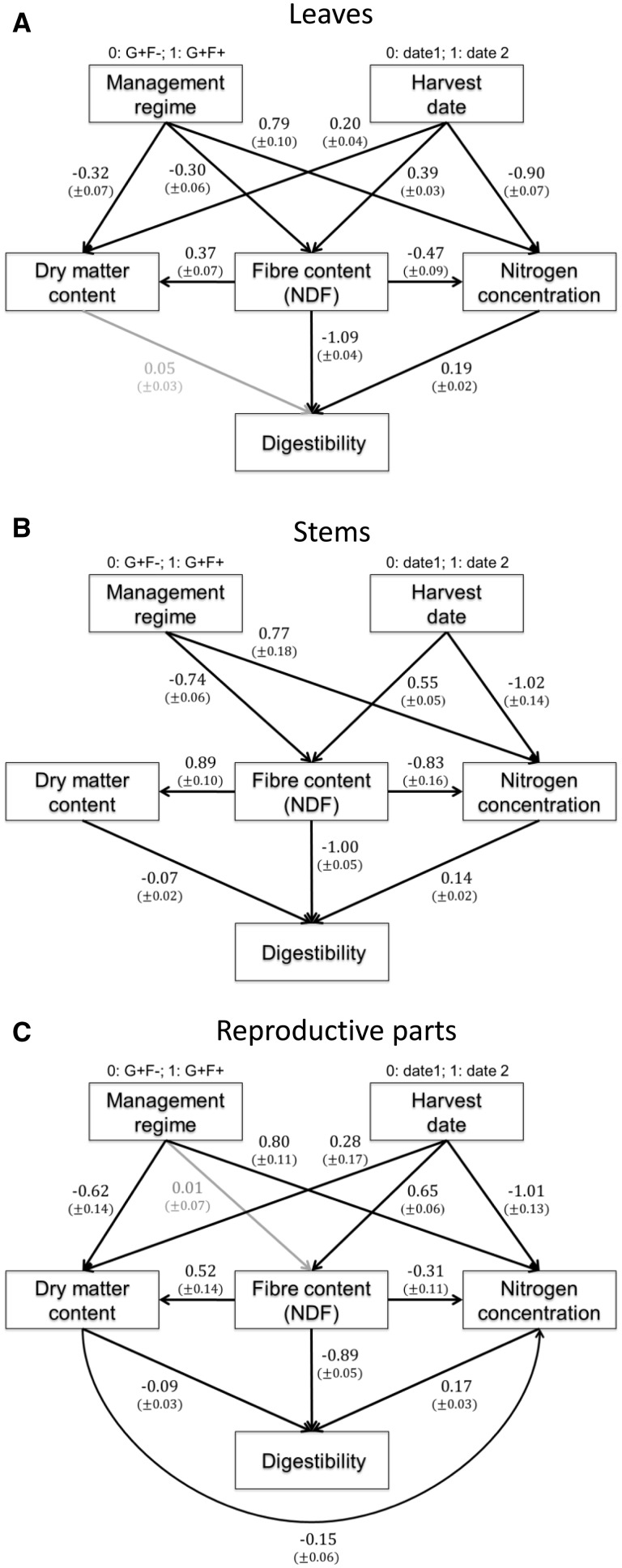
Structural equation models for (A) leaves, (B) stems and (C) reproductive parts. Path coefficients between variables are standardized partial regression coefficients. Intercepts are provided in brackets. Note that management regime and developmental stage are exogenous variables (no arrow point to them).

Harvest date negatively affected leaf NC and positively affected leaf NDF and DMC across all 16 plant species (Fig. 5A; Χ^2 ^=^ ^11.04, df = 8, *P *=* *0.20). Conversely, management regime induced an increase in leaf NC and a decrease in leaf NDF and DMC. An increase in NDF can induce an increase in DMC and a decrease in NC. Leaf DMD was influenced more by NDF than NC, and was not affected by DMC (path coefficient not significant) when considering the effect of NDF. For stems, management regime and harvest date affected only NDF and NC in the same direction as that for leaves. Variations in stem DMC were caused only by variations in NDF. DMD was principally influenced by NDF and slightly influenced by NC and DMC (Fig. 5B; Χ^2 ^=^ ^17.96, df = 12, *P *=* *0.12). The model retained for reproductive plant parts was similar to those for leaves and stems, except that NDF was not influenced by management regime (path coefficient not significant), and DMC influenced NC (Fig. 5C; Χ^2 ^=^ ^9.93, df = 6, *P *=* *0.13).

The indirect influence of harvest date was equivalent to management regime on leaf DMD (path coefficients were −0.61 and +0.48, respectively) and on stem DMD (path coefficients were −0.78 and +0.97, respectively). For reproductive parts, DMD was more influenced by harvest date than management regime (path coefficients were −0.86 and +0.21, respectively).

## Discussion

An initial objective of this study was to test the influence of plant species, harvest dates and management regimes on the forage quality of different plant parts. While most previous studies have considered mainly grass species (but see Cruz *et al.* 2002; Ammar *et al.* 2004), ours is amongst the first to have been conducted on a diversity of species belonging to different botanical families and growth forms. The trait-based approach followed here has allowed us (1) to assess some aspects of the functional response of this range of species to changes in the environmental conditions induced by the different management regimes, and (2) to relate this functional response to changes in forage digestibility, a key property of animal diet.

### Source of variation in plant digestibility

The range of values found here for leaf DMD (408-892 g  kg ^−^ ^1^) was larger than those obtained in previous studies, which ranged from 700 to 845 g kg ^−^ ^1^ for leaves of 14 grasses (Al Haj Khaled *et al.* 2006) and from 546 to 759 g kg ^−^ ^1^ for leaves of four functional types established for Poaceae (Duru *et al.* 2008*a*). The same trend occurred for stem DMD of these four functional types which varied from 421 to 699 g kg ^−^ ^1^ (Duru *et al.* 2008*a*). In our study, leaves generally had higher digestibility than stems and reproductive plant parts, which agrees with previous studies conducted on grass species only (Bidlack *et al.* 1999; Arzani *et al.* 2004; Karn *et al.* 2006; Duru *et al.* 2008*b*; Beecher *et al.* 2013) or both grass and dicotyledonous species (Duru 1997).

Forage quality varied significantly according to species growth form: grass species, composed mainly of tussocks, had the lowest DMD for each plant part. This was especially the case for *Stipa pennata*, the least consumed species (field observations), which also had the highest NDF and DMC and the lowest NC. Conversely, in agreement with Duru (1997), dicotyledonous species, composed of rosettes and extensive and stemmed-herbs, had the highest DMD. DMD variations across growth forms are explained by differences in NDF and DMC. Tussocks had higher NDF and DMC than rosettes and extensive and stemmed-herbs, as also found in previous studies (Cruz *et al.* 2002 for DMC; Buxton and Redfearn 1997 for NDF).

In agreement with our hypothesis, forage quality was significantly influenced by harvest dates. DMD and NC decreased while NDF increased with developmental stage for all species studied. These results agree with previous studies conducted on the species (Karn *et al.* 2006; Carrère *et al.* 2010; Asaadi and Yazdi 2011; Atis *et al.* 2012) and community levels (Pérez Corona *et al.* 1998; Bruinenberg *et al.* 2002; Garcia *et al.* 2003; Michaud *et al.* 2012). However, in most previous studies, these changes in forage quality were assessed at the whole-plant (or at the community level) (Carrère *et al.* 2010; Asaadi and Yazdi 2011) and were associated with changes in the proportion of different plant parts during development (Buxton 1996; Bruinenberg *et al.* 2002).

Here we analyzed the temporal changes in the quality of three plant parts—leaves, stems and reproductive parts—separately, and showed that digestibility decreased with time in all three. In leaves, this results from (1) the ageing process of individual leaves during which the concentrations of nitrogen and water-soluble carbohydrate decrease while that of fibre increases (Terry and Tilley 1964; Karn *et al.* 2006), (2) increase in the amount of indigestible cell walls per unit area with the level of insertion of the leaf on the shoot, at least in grasses (Groot and Neuteboom 1997). Similarly, in stems, there is an increase in cell-wall concentration and a decrease in soluble cells during development (Buxton 1996), making basal segments more digestible than upper segments (Pritchard *et al.* 1963). At the whole-plant and community levels, the decrease in digestibility with time, therefore results from a combination of an increase in the stem/leaf ratio of the shoot and a decrease in the digestibility of the different plant parts. Our study showed that in leaves, the decrease in digestibility was less pronounced for dicots than for grasses, which is in agreement with the finding that leaf cell-wall concentration varies less with time in legumes than in grasses (Jung and Allen 1995).

In the rangelands studied, the high abundance of tussocks (Bernard-Verdier *et al.* 2012; Barkaoui *et al.* 2013; Chollet *et al.* 2014) provide the amount of dry mass that sheep require, while rosette and extensive stemmed-herbs produce forage of higher quality over a longer period during spring. As shown in the study by Mládek *et al.* (2013), we assume that sheep recognize an ‘average forage value’: sheep maximize forage quality when forage is abundant and offers a choice of highly nutritious species, whereas when quality decreases, they maximize intake. Hassoun *et al.* (2007) found that in certain rangelands dry matter intake in fertilized and intensely grazed treatments is higher than that in non-fertilized and moderately grazed treatments.

Fertilization combined with more intense grazing induced higher DMD. This increase is explained by an increase in NC (Pontes *et al.* 2007; Carrère *et al.* 2010) and a decrease in fibre content (Duru and Ducrocq 2002; Carrère *et al.* 2010) and DMC (Pontes *et al.* 2007). Wilson *et al.* (1999) reported that the lower digestibility of species that prefer low-nutrient habitats is the consequence of their chemical composition and tissue anatomy, which is due to their high proportion of non-veinal sclerenchyma cells (Van Arendonk and Poorter 1994). Duru and Ducrocq (2002) found that the digestibility of the latest mature leaf and whole green lamina under severe defoliation was higher than that under low defoliation, and that the decrease in digestibility accelerated when combined with fertilization. Our results showed that an interaction occurred between harvest date and management regime, and the digestibility of different plant parts in the G+F+  treatment remained higher than that in the G+F− treatment at both harvest dates.

### Relationships between dry matter content and forage quality

DMC was strongly linked to DMD, NDF and NC, and slopes of these relationships were similar between harvest dates for every plant part. This finding indicates that variation in DMC induced a similar variation in DMD as a component of forage quality at both harvest dates. Intercepts generally differed between the two harvest dates. For a low DMC, DMD and NC were higher at the beginning of growth than at the biomass peak, while the opposite occurred for NDF. Comparison of the slopes of the relationships between DMD and DMC on the one hand and between DMD and the other components of forage quality on the other revealed that trait variations (DMD and components of forage quality) caused an identical range of variations in DMD of different plant parts (similar slopes). This pattern was not found for their intercepts which differed between the relationships. For a given DMC, leaves of the 16 species had higher DMD and NC than stems, while leaf NDF was the lowest, which validates our previous results for the range in DMD among plant parts. Measurement of the DMC of particular plant parts makes it possible to predict forage quality of each part at another harvest date and to predict the DMD of other plant parts.

Direct links were found between DMD, components of forage quality and DMC, especially between DMD and NDF. This relationship has been demonstrated in previous studies, but only as correlation and not in terms of causality. Previous studies found a negative correlation between DMD and DMC at the species level for Poaceae species (Al Haj Khaled *et al.* 2006; Pontes *et al.* 2007) and at the community level (Andueza *et al.* 2010; Gardarin *et al.* 2014). Our results confirm that DMC is a suitable indicator of DMD for leaves, stems and reproductive parts, even when variations in DMD are caused more by NDF and NC than by DMC. Variations in the digestibility of plant parts are caused mostly by structural (NDF) changes rather than chemical (NC) changes. We assume that the influence of NDF on DMD was particularly due to cellulose (Beni de Sousa *et al.* 1982) and lignin (Beni de Sousa *et al.* 1982; Jung and Allen 1995). The variation in DMC was partly due to fibre content, which has high structural carbohydrate content and a low proportion of mesophyll (Garnier and Laurent 1994). The increase in fibre content was responsible for the increase in DMC and consequently the decrease in DMD. Duru *et al.* (2008*b*) demonstrated that DMD was higher in species with lower leaf DMC at the leafy stage; we confirmed that this also occurs at the reproductive stage. 

### Relative influence of species, developmental stage and management regime on digestibility variation

Our approach is original in its use of path analysis to understand the relative influence of each factor on DMD. Among the factors studied, species developmental stage and management regime seemed to equally influence DMD of leaves and stems. In previous studies, developmental stage was shown as the most important factor affecting cell wall concentration and composition (Jung 2012) and thus digestibility (Buxton 1996).

The relative influence of species, harvest date and management regime on components of forage quality, leaf DMC and DMD was also assessed at the intraspecific level using the four species found in the two treatments. Harvest date and fertilization had the strongest effects on NC as demonstrated elsewhere (Kazakou *et al.* 2014), while species effects were strongest for NDF and DMC. As digestibility depends on a complex combination of NC, NDF, DMC (cf. Fig. 5), the relative effects of harvest date, management regimes and species on DMD were intermediate between the effects of these factors on NC on the one hand and NDF and DMC on the other hand. Although the influence of a number of factors on leaf digestibility has been assessed at the intraspecific level (Groot *et al.* 1999; Duru 2008; Nissinen *et al.* 2010), these have generally been done for each factor individually, making the comparison with our findings difficult. Only the study of Beni de Sousa *et al.* (1982) considered the relative importance of different fibre components on DMD and the leaf tensile strength of a grass species at the whole-plant level. Cellulose and lignin concentration had the highest influence on DMD, in agreement with our study where NDF, which takes into account two fibre components, was assessed.

## Conclusions

Better assessment of the nutritional value of forage in species-rich systems requires knowledge of their growth form composition. Developmental stage is an essential factor that induces changes in forage quality, which is also influenced by management regimes. Functional traits and especially DMC of the different plant parts, which is closely related to fibre concentration, is a good and easily measured predictor of DMD in these species-rich systems dominated by herbaceous species. Future studies could extend these results to vegetation including a wider range of growth forms, as our results revealed the importance of maintaining a complex community structure in rangelands (different species, growth forms and development stages) in order to maintain their nutritive value in time. A large range of variation in the DMC of the leaves has also been shown to improve the flexibility of production in permanent grasslands (Duru *et al.* 2014*b*). These various findings suggest that a high functional diversity of certain key traits might be required to enhance several aspects of forage production and quality.

## Sources of Funding

This work was funded by the EC2CO CASCADE (INSU CNRS) project. I.B. was supported by a doctoral fellowship from Montpellier Supagro and by a travel grant from ED SIBAGHE (Université Montpellier 2) to attend a course on path analysis in Sherbrook University given by B. Shipley.

## Contributions by the Authors

I.B., E.K., E.G. and J.R. designed the experiment. I.B. and J.R. contributed to data collection. I.B., L.B. and J.R. performed laboratory analysis. I.B. and E.K. performed the statistical analyses. All co-authors discussed and interpreted results prior to writing. I.B., E.K., E.G. and D.B. contributed to writing the manuscript.

## Conflict of Interest Statement 

None declared.

## Supplementary Material

Supplementary Data
