# Supplementary material for: Influence of management regime and harvest date on the forage quality of rangelands plants: the importance of dry matter content
Source: AoB Plants. 2016 Aug 2;8:plw045. doi: 10.1093/aobpla/plw045 (PMC4972474; doi:10.1093/aobpla/plw045)
Supplement: Supplementary Data [file supp_8_plw045_index.html]

Influence of management regime and harvest date on the forage quality of rangelands plants: the importance of dry matter content — Supplementary Data 

# Influence of management regime and harvest date on the forage quality of rangelands plants: the importance of dry matter content

## Supplementary Data

files

- Supplementary Data - zip file
