# Supplementary material for: Influence of management regime and harvest date on the forage quality of rangelands plants: the importance of dry matter content
Source: AoB Plants. 2016 Aug 2;8:plw045. doi: 10.1093/aobpla/plw045 (PMC4972474; doi:10.1093/aobpla/plw045)
Supplement: Supplementary Data [file supp_plw045_suppl_data.zip › aobplants-15201-T-s02.doc]

# OPEN ACCESS – RESEARCH ARTICLE

**Influence of management regime and harvest date on the forage quality of rangelands plants: the importance of dry matter content**

**Iris Bumb^1,*^, Eric Garnier^2^, Denis Bastianelli^3^, Jean Richarte^1^, Laurent Bonnal^3^ and Elena Kazakou^1^**

^1^Montpellier Supagro, Centre d’Ecologie Fonctionnelle et Evolutive (UMR CEFE), CNRS - Université de Montpellier - Université Paul-Valéry Montpellier - EPHE, campus CNRS, 1919 route de Mende, 34293 Montpellier cedex 5, France

^2^Centre d’Ecologie Fonctionnelle et Evolutive (UMR 5175), CNRS - Université de Montpellier - Université Paul-Valéry Montpellier - EPHE, campus CNRS, 1919 route de Mende, 34293 Montpellier cedex 5, France

^3^CIRAD, UMR SELMET, Baillarguet, 34398 Montpellier cedex 5, France

*Corresponding author

Corresponding author’s e-mail address: iris.bumb@cefe.cnrs.fr

Running head: Dry matter content predicts forage quality
