# Supplementary material for: Influence of management regime and harvest date on the forage quality of rangelands plants: the importance of dry matter content
Source: AoB Plants. 2016 Aug 2;8:plw045. doi: 10.1093/aobpla/plw045 (PMC4972474; doi:10.1093/aobpla/plw045)
Supplement: Supplementary Data [file supp_plw045_suppl_data.zip › aobplants-15201-T-s01.doc]

**File 1.**

|  |  | Number of samples | Mean | Standard deviation | Standard error (cross validation) | R² (cross validation) |
| --- | --- | --- | --- | --- | --- | --- |
| DMD, g.kg^-1^ | *In vitro* dry matter digestibility | 870 | 517 | 164 | 3.85 | 0.95 |
| NC, g.kg^-1^ | Total nitrogen | 1008 | 17.7 | 6.78 | 0.83 | 0.96 |
| NDF, %DM | Neutral detergent fibre | 1013 | 56.9 | 12.2 | 3.40 | 0.92 |

**File 2.**

|  | DMD of leaf | | | DMD of stem | | | DMD of reproductive parts | | |
| --- | --- | --- | --- | --- | --- | --- | --- | --- | --- |
|  | Harvest date 1 (n=20) | Harvest date 2 (n=20) | Total (n=40) | Harvest date 1 (n=6) | Harvest date 2 (n=17) | Total (n=23) | Harvest date 1 (n=8) | Harvest date 2 (n=18) | Total (n=26) |
| Nitrogen concentration  (NC) | **0.70***** | **0.79***** | 0.78*** | ***0.80*** | **0.54*** | 0.61** | 0.81* | 0.67** | 0.76*** |
| Fibre content (NDF) | -0.82*** | -0.89*** | -0.86*** | **-0.97***** | **-0.94***** | **-0.94***** | -0.60ns | -0.79*** | -0.79*** |
| Dry matter content (DMC) | **-0.90***** | **-0.91***** | -0.84*** | *-0.89* | **-0.85***** | -0.84*** | -0.46ns | -0.75*** | -0.73*** |

**File 3.**

|  | Dry Matter Digestibility (DMD) | | | Nitrogen Concentration (NC) | | | Fibre content (NDF) | | | Dry Matter Content (DMC) | | |
| --- | --- | --- | --- | --- | --- | --- | --- | --- | --- | --- | --- | --- |
|  | Leaf (n=89) | Stem (n=54) | Repro. parts (n=57) | Leaf (n=89) | Stem (n=54) | Repro. parts (n=57) | Leaf (n=89) | Stem (n=54) | Repro. parts (n=57) | Leaf (n=89) | Stem (n=57) | Repro. parts (n=57) |
| Harvest date | 255*** | 474*** | 193*** | 262*** | 39.2*** | 46.6*** | 249*** | 712*** | 357*** | 226*** | 26.0*** | 20.5*** |
| Species | 90.5*** | 183*** | 138*** | 7.66*** | 4.19* | 4.12* | 637*** | 470*** | 446*** | 205*** | 35.7*** | 26.4*** |
| Management regime | 35.7*** | 179*** | ns | 175*** | 84.8*** | 47.1*** | 13.7*** | 33.7*** | 93.6*** | 32.7*** | 69.7*** | 9.17** |
| Harvest date × Management regime | 39.6*** | ns |  | 20.5*** | 7.86** |  | 50.4*** | ns | 8.86** | 4.16* |  | 6.05* |
| Harvest date × Species | 37.8*** |  |  | 13.3*** |  |  | 43.4*** |  |  | 5.31** |  |  |
| Management regime × Species |  | 18.0*** |  |  |  |  | ns | 8.86*** | 3.22* |  | 22.2*** | 2.88* |
